# Supplementary figures and images for: Proteomic Study of Response to Copper, Cadmium, and Chrome Ion Stress in Yarrowia lipolytica Strains Isolated from Andean Mine Tailings in Peru
Source: Microorganisms. 2022 Oct 11;10(10):2002. doi: 10.3390/microorganisms10102002 (PMC9611812; doi:10.3390/microorganisms10102002)

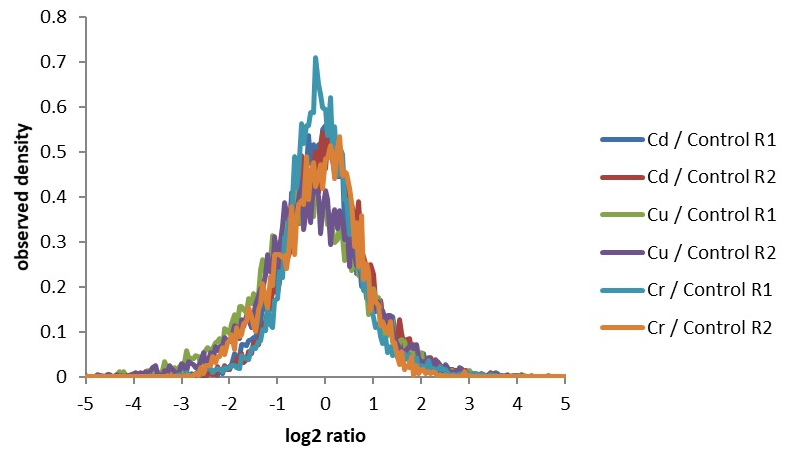

Supplement: Supplementary file 1 [file microorganisms-10-02002-s001.zip › Figure S1. Normalized Treatment-Control reporter ion ratios.png]
